# Supplementary material for: Genomic Characterization of Endemic and Ecdemic Non-typhoidal Salmonella enterica Lineages Circulating Among Animals and Animal Products in South Africa
Source: Front Microbiol. 2021 Oct 4;12:748611. doi: 10.3389/fmicb.2021.748611 (PMC8521152; doi:10.3389/fmicb.2021.748611)
Supplement: Supplementary file 1 [file Data_Sheet_1.zip › Supplementary Figures.PDF]

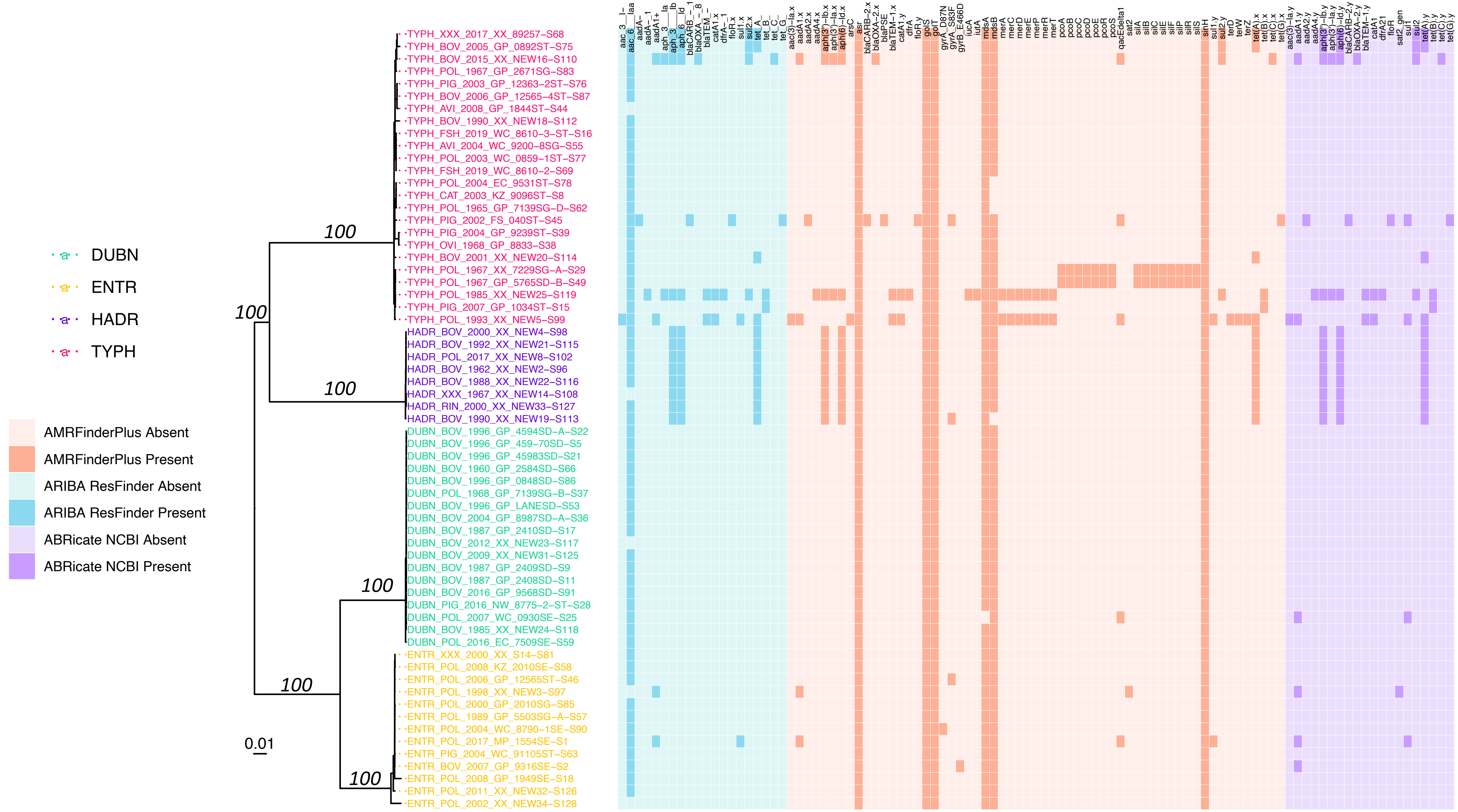

Supplementary Figure S1. Maximum likelihood phylogeny constructed using core SNPs identified among the genomes of 63 *Salmonella* strains isolated in conjunction with this study. Tip label colors denote isolate serotypes, and branch labels denote ultrafast bootstrap support percentages out of 1,000 replicates (selected for readability). The heatmap to the right of the phylogeny denotes the presence and absence of antimicrobial resistance (AMR) and/or stress response determinants detected in each genome using (i) ARIBA + the ResFinder AMR database (blue), (ii) AMRFinderPlus (orange), and (iii) ABRicate + the NCBI AMR database (purple). The phylogeny is rooted at the midpoint with branch lengths reported in substitutions per site. Core SNPs were identified among all genomes using kSNP3. AMR determinants identified using ABRicate were considered to be present in a genome at minimum identity and coverage thresholds of 75 and 50%, respectively. The phylogeny was constructed and annotated using IQ-TREE and bactaxR/ggtree, respectively.

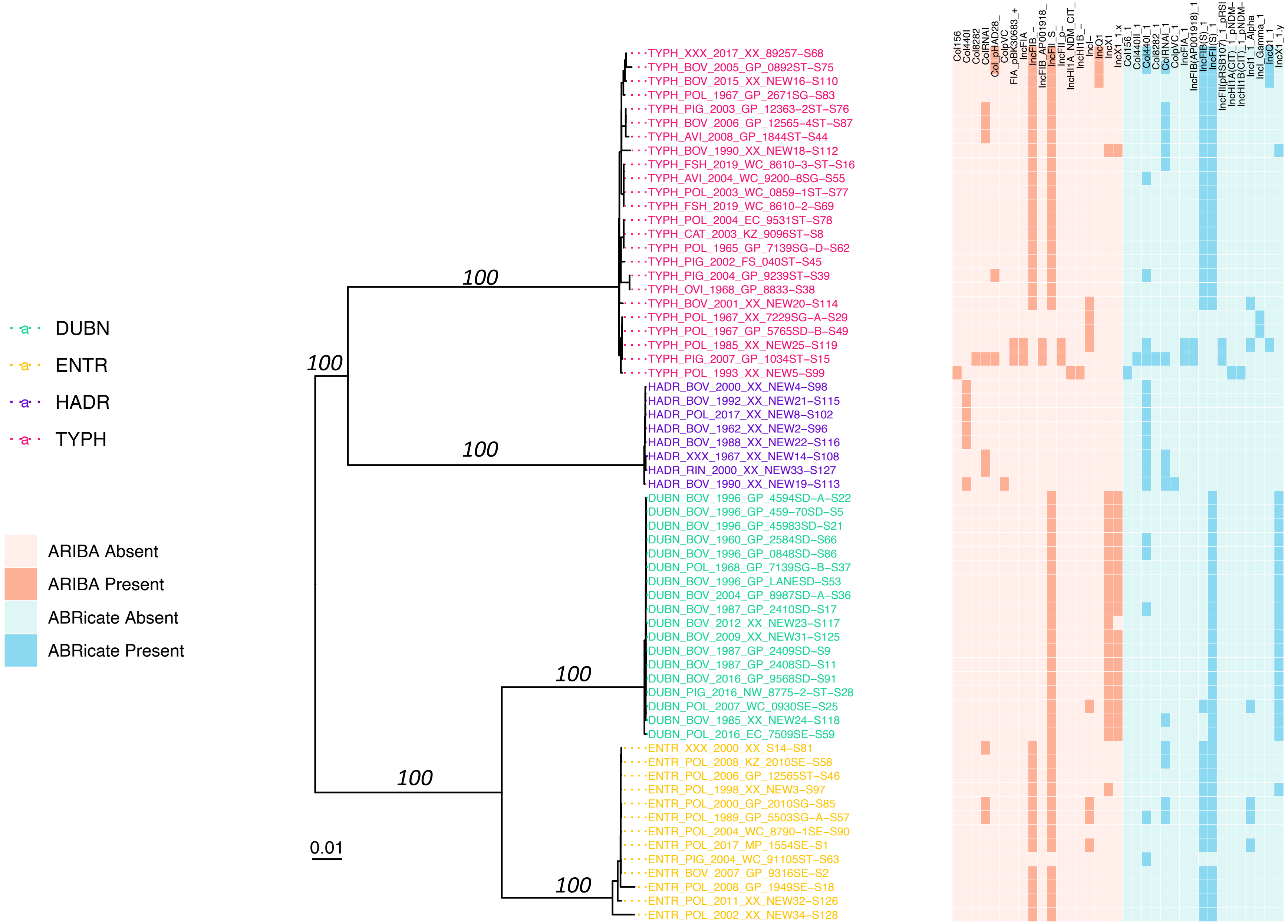

Supplementary Figure S2. Maximum likelihood phylogeny constructed using core SNPs identified among the genomes of 63 *Salmonella* strains isolated in conjunction with this study. Tip label colors denote isolate serotypes, and branch labels denote ultrafast bootstrap support percentages out of 1,000 replicates (selected for readability). The heatmap to the right of the phylogeny denotes the presence and absence of plasmid replicons detected in each genome using the PlasmidFinder database and (i) ARIBA (orange), and (ii) ABRicate (blue). The phylogeny is rooted at the midpoint with branch lengths reported in substitutions per site. Core SNPs were identified among all genomes using kSNP3. Plasmid replicons identified using ABRicate were considered to be present in a genome at minimum identity and coverage thresholds of 80 and 60%, respectively. The phylogeny was constructed and annotated using IQ-TREE and bactaxR/ggtree, respectively.

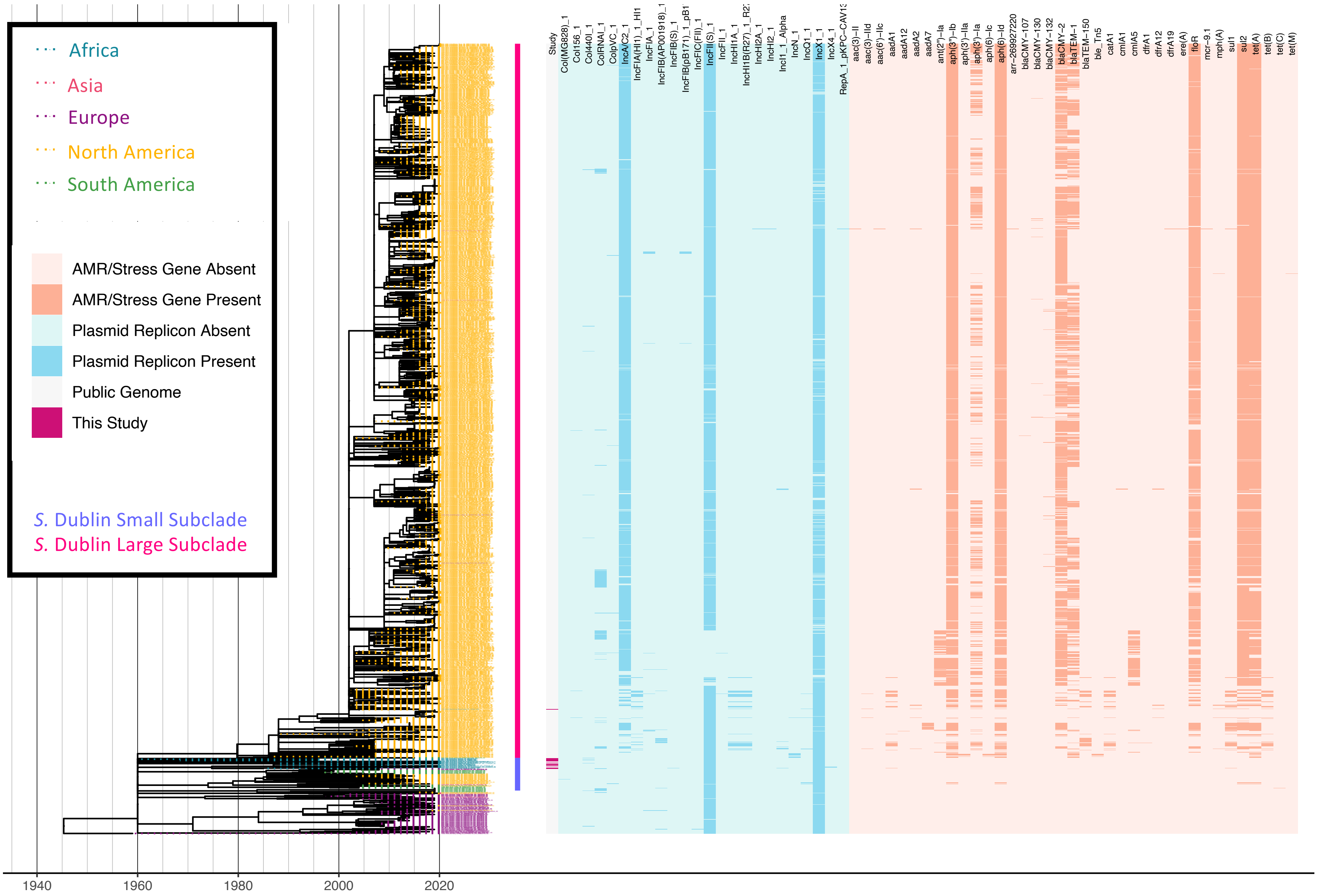

Supplementary Figure S3. Maximum likelihood phylogeny constructed using core SNPs identified among 1,787 *S.* Dublin genomes within *S.* Dublin Major Clade I (1,769 publicly available genomes, plus 18 sequenced here). Tip label colors denote the continent from which each strain was reported to have been isolated. Clade labels denote subclades assigned in this study and are shown to the right of tip labels. The heatmap to the right of the phylogeny denotes: (i) whether an isolate was sequenced in conjunction with this study (dark pink) or not (gray; "Study"); the presence and absence of (ii) plasmid replicons (blue) and (iii) antimicrobial resistance (AMR) determinants (orange). The phylogeny was rooted and time-scaled using LSD2, with branch lengths reported in years (X-axis). Core SNPs were identified among all genomes using Parsnp. AMR determinants were identified using ABRicate, the NCBI AMR determinant database, and minimum identity and coverage thresholds of 75 and 50%, respectively. Plasmid replicons were identified using ABRicate and the PlasmidFinder database, using minimum identity and coverage thresholds of 80 and 60%, respectively. The phylogeny was constructed and annotated using IQ-TREE and bactaxR/ggtree, respectively.

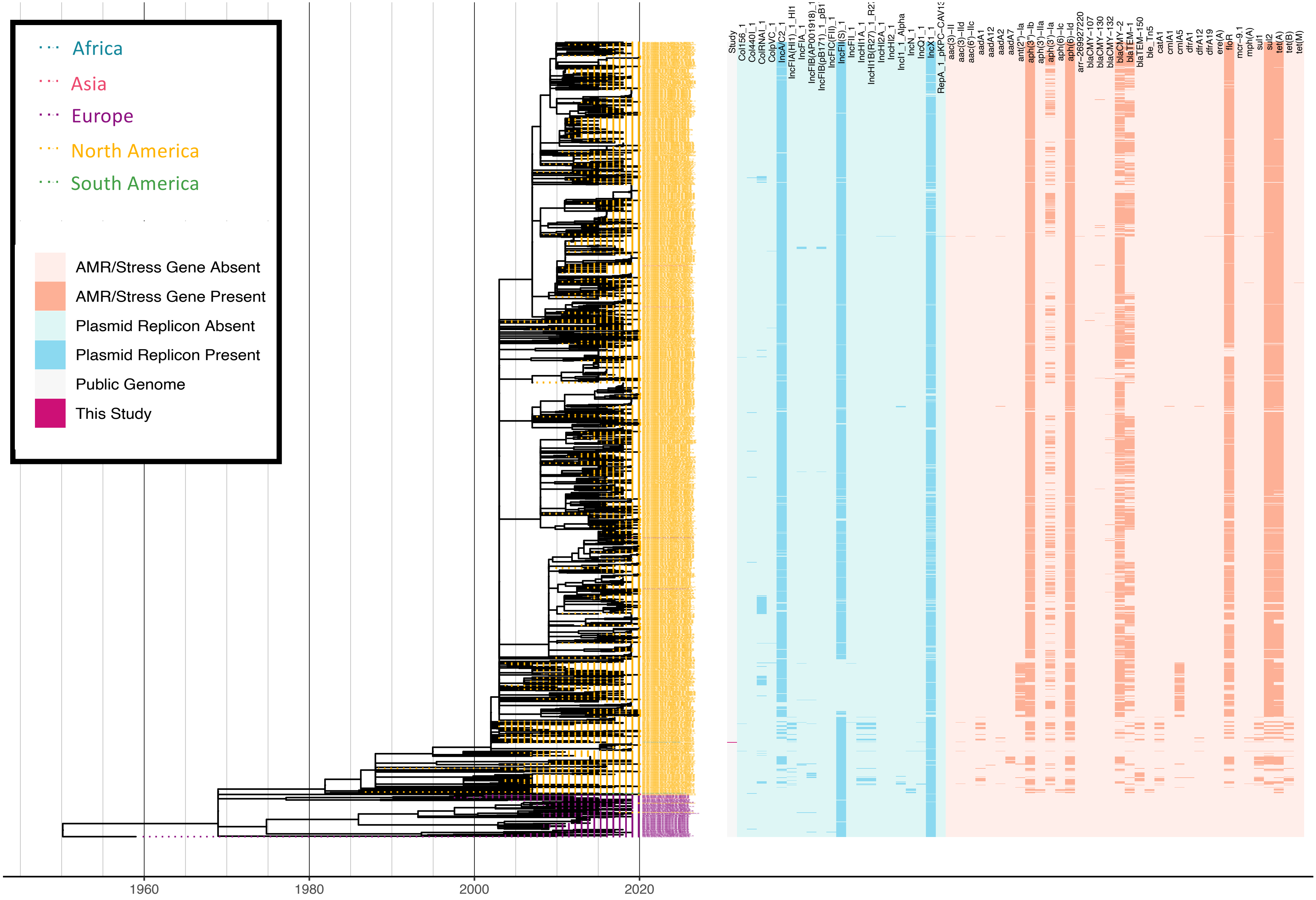

Supplementary Figure S4. Maximum likelihood phylogeny constructed using core SNPs identified among 1,709 *S.* Dublin genomes within the *S.* Dublin Large Subclade (1,708 publicly available genomes, plus one sequenced here). Tip label colors denote the continent from which each strain was reported to have been isolated. The heatmap to the right of the phylogeny denotes: (i) whether an isolate was sequenced in conjunction with this study (dark pink) or not (gray; "Study"); the presence and absence of (ii) plasmid replicons (blue) and (iii) antimicrobial resistance (AMR) determinants (orange). The phylogeny was rooted and time-scaled using LSD2, with branch lengths reported in years (X-axis). Core SNPs were identified among all genomes using Parsnp. AMR determinants were identified using ABRicate, the NCBI AMR determinant database, and minimum identity and coverage thresholds of 75 and 50%, respectively. Plasmid replicons were identified using ABRicate and the PlasmidFinder database, using minimum identity and coverage thresholds of 80 and 60%, respectively. The phylogeny was constructed and annotated using IQ-TREE and bactaxR/ggtree, respectively.

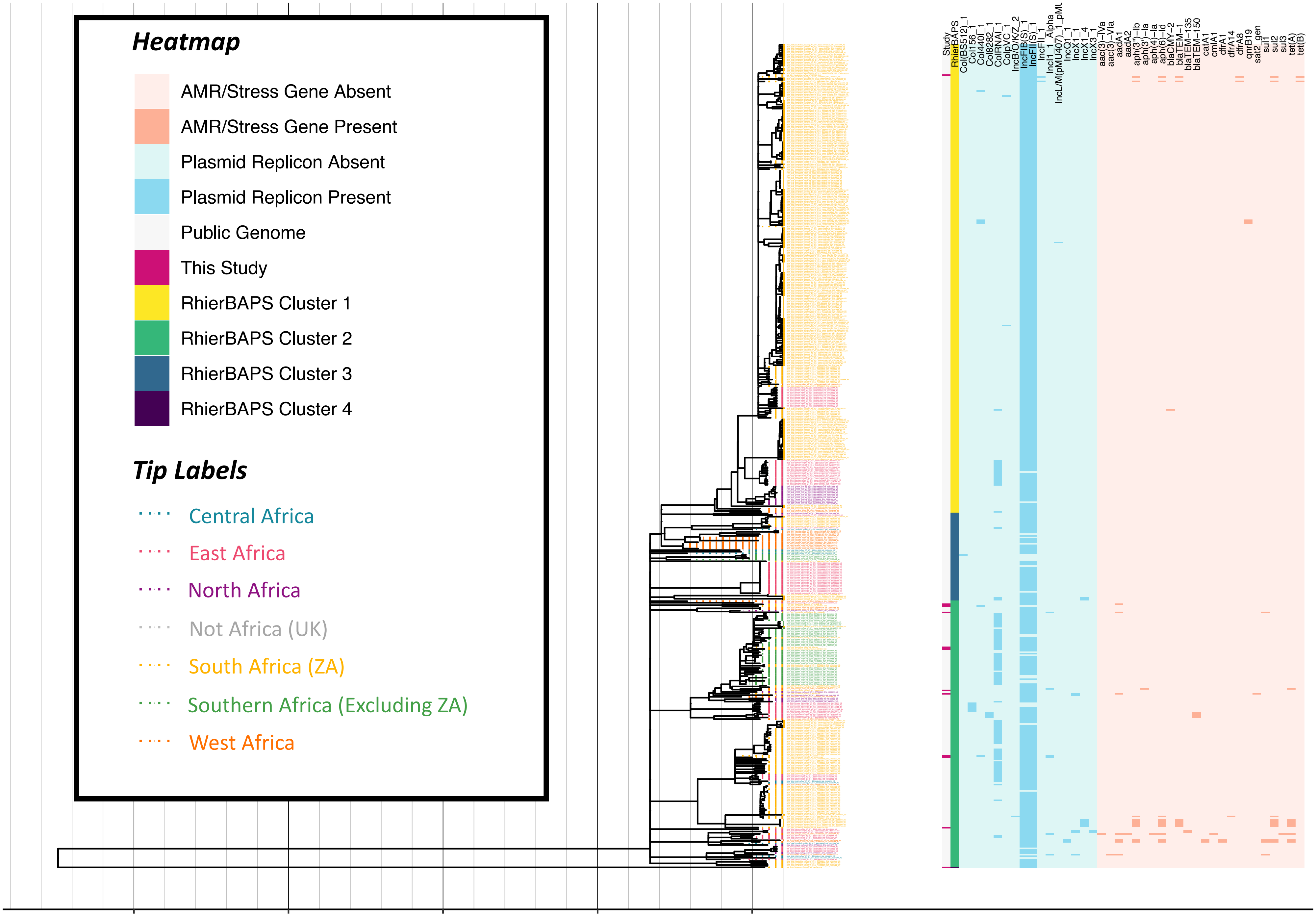

Supplementary Figure S5. Maximum likelihood phylogeny constructed using core SNPs identified among 517 ST11 *S.* Enteritidis genomes (505 publicly available genomes, plus 12 sequenced here). Tip label colors denote the region/country from which each strain was reported to have been isolated (based on African regions as defined by the African Union, 25 April 2021). The heatmap to the right of the phylogeny denotes: (i) whether an isolate was sequenced in conjunction with this study (dark pink) or not (gray; "Study"); (ii) level 1 cluster assignments obtained using RhierBAPS ("RhierBAPS"); the presence and absence of (iii) plasmid replicons (blue) and (iv) antimicrobial resistance (AMR) determinants (orange). The phylogeny was rooted and time-scaled using LSD2, with branch lengths reported in years (X-axis). Core SNPs were identified among all genomes using Parsnp. AMR determinants were identified using ABRicate, the NCBI AMR determinant database, and minimum identity and coverage thresholds of 75 and 50%, respectively. Plasmid replicons were identified using ABRicate and the PlasmidFinder database, using minimum identity and coverage thresholds of 80 and 60%, respectively. The phylogeny was constructed and annotated using IQ-TREE and bactaxR/ggtree, respectively.

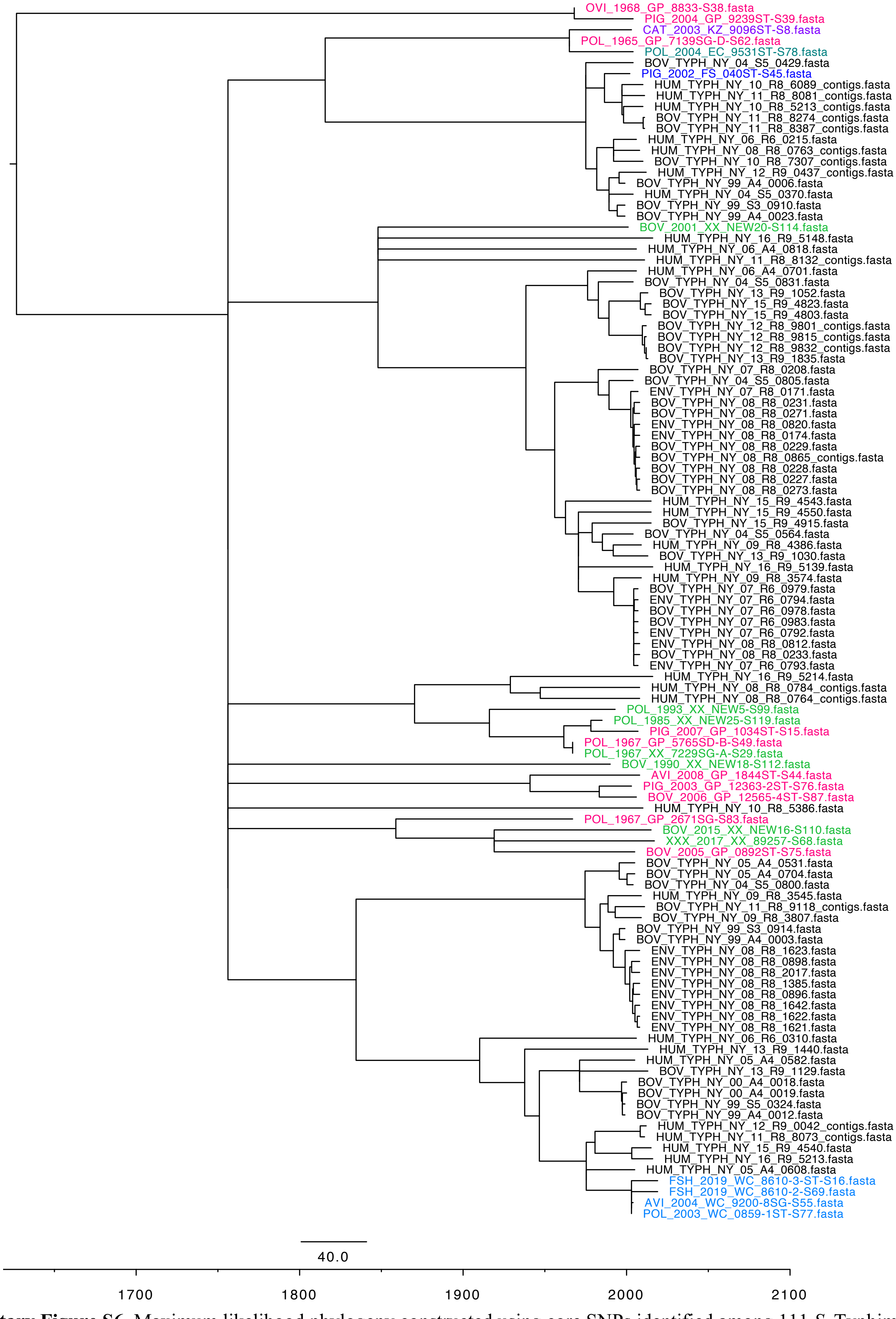

Supplementary Figure S6. Maximum likelihood phylogeny constructed using core SNPs identified among 111 *S.* Typhimurium genomes (87 publicly available genomes from a previous study of human- and bovine-associated *S.* Typhimurium in New York State, plus 24 sequenced here). Publicly available genomes from New York State (United States of America) are denoted by black tip labels, while genomes sequenced here are denoted by colored tip labels corresponding to the province from which their associated strains were isolated. The phylogeny was rooted and time-scaled using LSD2, with branch lengths reported in years (X-axis). Core SNPs were identified among all genomes using Parsnp. The phylogeny was constructed and annotated using IQ-TREE and FigTree, respectively.
